# Crescentic Glomerulonephritis with Anti-GBM and p-ANCA Antibodies

**DOI:** 10.1155/2012/132085

**Published:** 2012-02-26

**Authors:** Tariq Javed, Parag Vohra

**Affiliations:** Department of Medicine, Bakersfield Memorial Hospital, Bakersfield, CA 93301, USA

## Abstract

We are presenting a case of renal failure with anti-GBM and p-ANCA antibodies positive. Patients with dual antibodies are considered to be a vasculitis-variant of anti-GBM antibody nephritis. These patients may have atypical presentation and it may delay diagnosis and treatment. Recurrence rate is higher in these patients. We reviewed the literature of cases and studies on cresenteric glomerulonephritis with anti-GBM and p-ANCA positive patients. We recommend that patients suspected with pulmonary-renal syndrome should be checked for anti-GBM and p-ANCA antibodies, should undergo renal biopsy and should should have close long term follow up to watch for recurrence.

## 1. Introduction

In 20–25% of patients with antiglomerular basement membrane GBM nephritis, p-ANCA is also present [1, 2]. Patients with dual antibodies are considered to be a vasculitis-variant of anti-GBM antibody nephritis. Such patients may not have a typical presentation of pulmonary-renal syndrome, resulting in delay of the correct diagnosis and initiation of treatment [1–3]. Anti-GBM nephritis usually is a monophasic disease which rarely recurs [4, 5]. The risk of recurrence is higher in patients with persistently elevated ANCA levels following resolution of the acute episode [2, 4]. Such patients require frequent followup, long-term maintenance immunosuppressive treatment, and reinitiation of induction therapy including plasmapheresis if the disease recurs with rapidly progressive glomerulonephritis [1, 3, 6]. Awareness of atypical presentations, the risk of recurrence, and overlap between ANCA vasculitis and anti-GBM nephritis is critical for early diagnosis, appropriate treatment, and improved renal outcome in these patients. Herein, we present a 57 years old Caucasian female with anti-GBM nephritis who presented with vasculitis symptoms and additionally had positive p-ANCA titers.

## 2. Case Report

A 57-year-old Caucasian female with a history of hypertension came to the emergency department (ED) for evaluation of worsening nonproductive cough and exertional dyspnea for 2 weeks. She had gone to her primary care physician for the above-mentioned symptoms and was given cephalexin. Despite completing the course of antibiotic, her symptoms progressed. She developed a diffuse body rash 2 days after starting cephalexin and complained of diffuse joint pain and malaise for 2 weeks. She denied having abdominal pain, fever, or hemoptysis and was not using any other nephrotoxic drugs including over the counter medications. Laboratory studies performed by the primary care physician 2 weeks prior, to presentation, including renal function tests, were unremarkable.

Vital signs in the ED were BP: 132/86, P: 118, R/R: 20, and afebrile. Oxygen saturation was 94% on 2 L/min nasal cannula and lung auscultation was normal. She had a diffuse maculopapular rash on the anterior chest wall, trunk area, and all extremities. Laboratory results showed sodium 127 mmol/L (132–150 mmol/L), potassium 3.8 mmol/L (3.5–5.5 mmol/L), bicarbonate 22.7 mmol/L (23–31 mmol/L), BUN 38 mg/dL (5–23 mg/dL), creatinine 4.39 mg/dL (0.44–1.03 mg/dL), chloride 93 mmol/dL (91–110 mmol/dL), calcium 8.3 mg/dL (8.7–10.2 mg/dL), and anion gap 9.3 (3–11). Urine analysis at admission showed specific gravity 1.006 (1.010–1.025), blood 3+, ph 5 (4.5–8.5), protein 1+, RBC ≥ 100, WBC 0–5, negative nitrites, negative leukocyte esterase, and no casts. WBC count at admission was 8400 with no shift, and hemoglobin and hematocrit were 8.8 and 25.8, respectively. Chest X-ray showed no acute pulmonary pathology. At the time of admission, the differential diagnoses were (1) drug-induced interstitial nephritis, (2) postinfectious glomerulonephritis, (3) acute tubular necrosis, and (4) pulmonary-renal syndrome including Goodpasture's syndrome, microscopic polyangiitis, or Wegener's granulomatosis.

Cephalexin was stopped and she was started on IV hydration but responded poorly and remained oliguric. Additional laboratory studies revealed negative urine eosinophils, normal C3 and C4 levels, and negative ANA, rheumatoid factor, and ASO titers. ANCA at admission was positive at ≥1 : 20 with a perinuclear pattern, confirmed as MPO (myelo-peroxidase) ANCA on ELISA. Anti-GBM IgG antibody was positive with titer 234 au/mL (0–19 au/mL). Repeated chest X-ray on day 4 and day 5 revealed development of bilateral alveolar infiltrates. She underwent bronchoscopy which showed evidence of alveolar hemorrhage, and lung biopsy which disclosed acute fibrinous and interstitial pneumonia with interstitial neutrophils but no granulomas. Renal biopsy also was performed.

## 3. Kidney Biopsy

**Figure 1:**
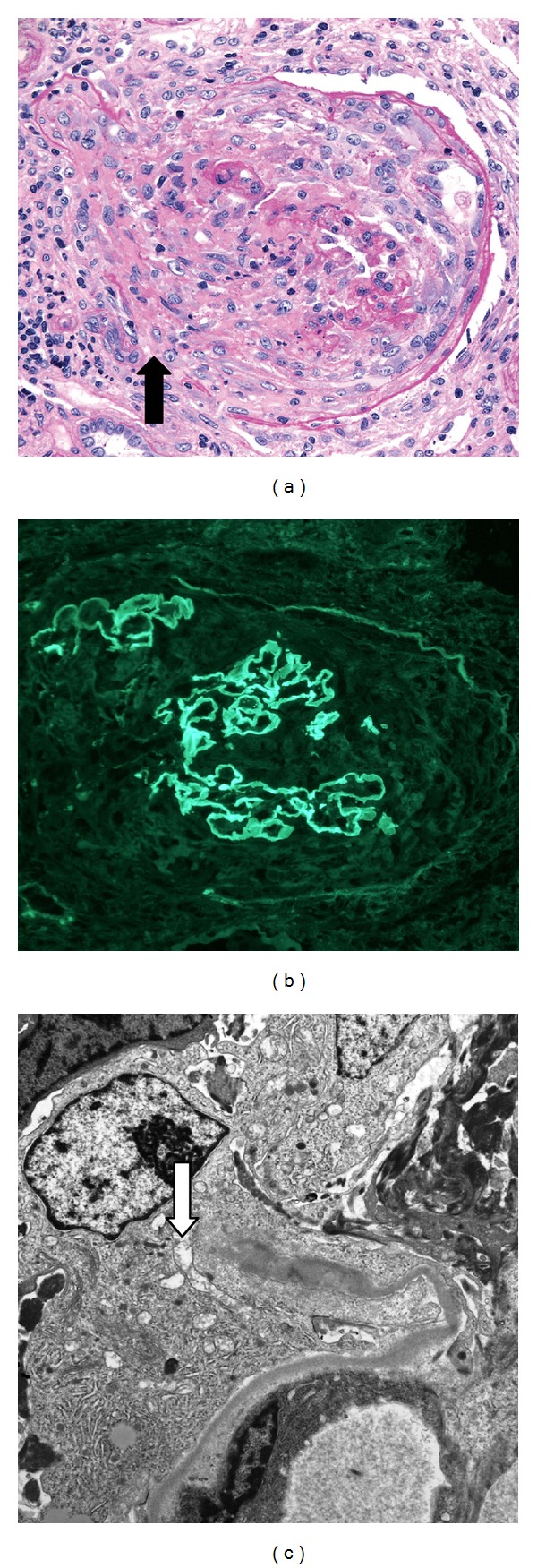
Glomeruli from the renal biopsy. (a) Cellular destructive crescent with discontinuities in Bowman's capsule (arrow). The adjacent interstitium had edema and a mononuclear leukocytic infiltrate. Periodic acid-Schiff ×250. (b) Immunofluorescence showing strong (3-4+) linear IgG staining along capillary walls. The capillary walls are disrupted focally due to crescent formation ×250. (c) Broken glomerular capillary wall (arrow) associated with a cellular crescent. There are no capillary wall electron dense deposits ×10,000.

The specimen for light microscopy contained renal cortex with 14 glomeruli, three of which were globally sclerotic. Eight glomeruli had crescents, all in an active stage (Figure 1(a)). Glomeruli with crescents frequently showed disruption of Bowman's capsule with fibrin and cells extending to the adjacent interstitium. There were no mesangial hypercellularity or segments of sclerosis. Tubular cells were necrotic, tubular lumina contained erythrocytes, and the interstitium was edematous with a lymphocytic infiltrate. Arteries had intimal fibrosis, but there was no vascular inflammation or necrosis. Immunofluorescence was performed on 14 glomeruli, all of which had strong linear capillary wall staining for IgG with lesser staining for kappa and lambda light chains (Figure 1(b)). All glomeruli also had urinary space staining for fibrin within crescents, and moderate granular mesangial staining for C3. There was no fibrin in vascular walls nor was there evidence of glomerular immune complex deposition. The specimen for electron microscopy contained 12 glomeruli, all of which had active crescents without mesangial hypercellularity. Ultrastructural study was performed on three glomeruli and confirmed disruptions of capillary and Bowman's capsular basement membranes (Figure 1(c)). There were no electron dense immune complex deposits or tubuloreticular structures.

## 4. Diagnosis

 Anti-GBM antibody nephritis with 85% active crescents by renal biopsy, and ANCA-positive vasculitis (microscopic polyangiitis) clinically.

### 4.1. Clinical Followup

She was started on cyclophosphamide (3 mg/kg daily), high-dose prednisone (1 mg/kg), and plasmapheresis, initially with 4 liters exchange plasmapheresis daily for the first week then every other day for 2 additional weeks. Followup anti-GBM antibody titers after the first, second, and third weeks of treatment were negative. ANCA levels also were negative after the first two weeks of plasmapheresis. Her pulmonary symptoms improved significantly; however, renal function remained impaired and dialysis was begun with continued dialysis dependence. She received maintenance doses of cyclophosphamide and prednisone for 3 months, with negative anti-GBM and ANCA titers and no clinical recurrence after 14 months of followup.

## 5. Discussion

 Goodpasture's syndrome is a rare clinical entity with a prevalence of less than 1 case per million population [7]. It is a cause of rapidly progressive glomerulonephritis characterized by pulmonary hemorrhage, glomerulonephritis, and elevated titers of anti-GBM antibody. The pathophysiological hallmark is formation of antibody binding to the noncollagenous domain of type IV collagen in the glomerular basement membrane, specifically an antigen on the alpha 3 chain [8–10]. This represents a classical type 2 immunological reaction, most commonly with antibodies of the IgG class, although IgA and IgM subtypes have been reported infrequently. Detection of anti-GBM antibody is done by ELISA and confirmed by western blot assay [11]. Up to 80% of patients have Goodpasture's syndrome, with both lung and renal involvement. Pulmonary injury is due to binding of the anti-GBM antibody to the alveolar capillary basement membrane, with alveolar hemorrhage in approximately 70% of cases, resulting in hemoptysis. Other pulmonary symptoms may include cough and dyspnea, and chest X-ray discloses pulmonary infiltrates. Patients with known lung injury due to smoking, cocaine abuse, or hydrocarbon exposure will have more extensive pulmonary damage [12]. Renal manifestations include acute renal failure with urinary findings of hematuria, nonnephrotic range proteinuria, dysmorphic red cells, and red cell and granular casts. Constitutional symptoms, such as malaise, weight loss, fever, or arthralgias, are typically absent. The presence of such symptoms suggests that the patient may have an associated vasculitis [1, 3]. The diagnosis requires identification of- anti GBM antibodies either in the serum or the kidney. Renal biopsy should be performed in all cases as the biopsy findings are useful both for establishing the diagnosis and to determine the extent of acute and chronic renal damage allowing for optimal patient management [13, 14].

 An important determinant of outcome is the initial serum creatinine concentration and the percentage of glomeruli involved with crescents. Renal outcome is poor if the serum creatinine is more than 5.7 mg/dL or more than 80% of glomeruli have crescents on renal biopsy at the time of diagnosis. Plasmapheresis in combination of prednisone and cyclophosphamide is the treatment of choice. Plasmapheresis is useful even for patients with a serum creatinine above 5.7 mg/dL and extensive glomerular damage [14]. Such patients have shown improvement in extra renal symptoms, specifically hemoptysis and respiratory failure which improve overall patient survival.

 All patients with anti-GBM antibody nephritis should be tested for ANCA. As mentioned above, 20–25% of patients with anti-GBM nephritis also have ANCA antibodies [1, 2, 15], known as WHO type IV crescentic disease. The majority of reported cases with dual antibodies are 50 to 80 years of age, which corresponds to the typical age of patients with ANCA-associated disease. In contrast, anti-GBM nephritis has a bimodal distribution, typically occurring in males in their 20 s and women above 80 years [7] old. Previous studies have noticed a prevalence of anti-GBM antibodies in 5% of ANCA vasculitis cases [6].Therefore, it is possible that many elderly patients with anti-GBM nephritis (second peak of bimodal curve) may in fact have ANCA vasculitis with superimposed anti-GBM antibody nephritis, as ANCA vasculitis is signficantly more common. One pathophysiological hypothesis regarding anti-GBM antibody formation is the p-ANCA injures of the glomerular basement membrane during crescent formation, exposing *de novo* basement membrane antigens and initiating development of an anti-GBM antibody [16]. *In vivo* experiments have demonstrated that MPO-ANCA can severely aggravate subclinical anti-GBM glomerular disease in rats [16]. It is unknown why only a small percentage of ANCA vasculitis patients develop anti-GBM antibodies, but this may relate to associated factors including the presence of underlying renal disease, smoking, infection, or environmental and genetic factors.

Patients with dual antibodies may have an atypical presentation, which may cause delay in the correct diagnosis and initiation of treatment. Some clinical features of vasculitis including arthralgias, rash, malaise, and fever may occur in these patients, while typically are absent in classic anti-GBM nephritis [1, 3]. Patients with persistence of ANCA after resolution of the initial symptoms have a higher incidence of recurrence and need close monitoring of ANCA levels [17]. Unlike classic anti-GBM cases, such patients should be treated with long-term maintenance immunosuppression such as azathioprine, methotrexate, or low-dose prednisone [6]. Our patient had vasculitis symptoms at presentation including malaise, arthralgias, and a maculopapular rash. However, the concurrent use of antibiotics and lack of pulmonary symptoms are presentation confusing the clinical picture, raising other entities in the differential diagnosis. She initially was treated for interstitial nephritis/acute tubular necrosis while awaiting the serologic tests. Specific treatment for anti-GBM nephritis was started on day 5, at which time the creatinine level was 5.9. It is possible she initially had ANCA vasculitis as her presenting vasculitic symptoms, age, absence of hemoptysis, and normal lung exam and pulmonary imaging were more suggestive of vasculitis. She subsequently may have developed anti-GBM antibodies with progressive renal failure, as the crescents were active and all in the same stage, a typical feature of anti-GBM antibody nephritis. The delay in initiation of immunosuppressive therapy may have allowed permanent renal damage, as the serum creatinine rose to 5.9 before the diagnosis was made. Had she been started on immunosuppressive treatment and plasmapheresis earlier, she might have had improved renal outcome.

## 6. Teaching Points

1. Patients with suspected pulmonary-renal syndrome should be tested for both ANCA and anti-GBM antibodies regardless of the other clinical symptoms.
2. Patients with anti-GBM antibody nephritis may have an atypical presentation, particularly elderly females. If the symptoms include rash, malaise, arthralgias, fever, and other constitutional ailments, associated vasculitis should be considered.
3. It is important to make the diagnosis and initiate treatment when the serum creatinine level is less than 6.0 mg/dL for optimal outcome and prognosis.
4. Renal biopsy should be performed early in course of disease, to confirm the diagnosis and guide patient management.
5. The risk of recurrence risk is high in patients with persistently elevated ANCA following resolution of an acute episode. Such patients require close followup and need long-term maintenance immune suppression.
